# Construction of Immune-Related ceRNA Network in Dilated Cardiomyopathy: Based on Sex Differences

**DOI:** 10.3389/fgene.2022.882324

**Published:** 2022-06-08

**Authors:** Chang Liu, Jian Liu, Daihong Wu, Shaoling Luo, Weijie Li, Lushan Chen, Zhen Liu, Bingbo Yu

**Affiliations:** ^1^ Department of Cardiology, Guangzhou First People’s Hospital, School of Medicine, South China University of Technology, Guangzhou, China; ^2^ Department of Cardiology, Guangzhou First People’s Hospital, Guangzhou Medical University, Guangzhou, China

**Keywords:** dilated cardiomyopathy, immunotherapy, sex differences, ceRNA network, gene regulation, bioinformatics

## Abstract

**Background:** Immune targeted therapy has become an attractive therapeutic approach for patients with dilated cardiomyopathy (DCM) recently. Genetic predisposition and gender play a critical role in immune-related responses of DCM. This study aimed to perform a bioinformatics analysis of molecular differences between male and female samples and identify immune-related ceRNA network in DCM.

**Methods:** The gene expression microarray and clinical features dataset of GSE19303 was downloaded from the GEO. The raw data were preprocessed, followed by identification of differentially expressed genes (DEGs) between male and female DCM samples. Crucial functions and pathway enrichment analysis of DEGs were investigated through GO analysis and KEGG pathway analysis, respectively. A lncRNA–miRNA–mRNA network was constructed and a central module was extracted from the ceRNA network.

**Results:** Compared with the female group, the male group benefits more from IA/IgG immunotherapy. Male patients of DCM had a significant positive correlation with the abundance of inflammatory cells (B cells, memory B cells, CD8^+^ Tem cells, and NK cells). Sex difference DEGs had a widespread impact on the signaling transduction, transcriptional regulation, and metabolism in DCM. Subsequently, we constructed an immune-related ceRNA network based on sex differences in DCM, including five lncRNAs, six miRNAs, and 29 mRNAs. Furthermore, we extracted a central module from the ceRNA network, including two lncRNAs (XIST and LINC00632), three miRNAs (miR-1-3p, miR-17-5p, and miR-22-3p), and six mRNAs (CBL, CXCL12, ESR1, IGF1R, IL6ST, and STC1). Among these DEGs, CBL, CXCL12, and IL6ST expression was considered to be associated with inflammatory cell infiltration in DCM.

**Conclusions:** The identified ceRNA network and their enriched pathways may provide genetic insights into the phenotypic diversity of female and male patients with DCM and may provide a basis for development of sex-related individualization of immunotherapy.

## Introduction

Globally, dilated cardiomyopathy (DCM) is one of the most common forms of cardiomyopathy, and it represents a leading cause of cardiac transplantation in children and adults (Martinez et al., 2021). Contemporary estimates of the DCM prevalence range from one in 2,500 to one in 250 people (Merlo et al., 2018). Theoretically, DCM is a heart muscle disease characterized by left or biventricular dilatation and systolic dysfunction in the absence of coronary artery disease, hypertension, valvular disease, or congenital heart disease (WJ et al., 2017). The important feature of DCM is the structural or functional abnormalities of the heart muscle, which leads to complications such as heart failure and arrhythmia and results in substantial morbidity and mortality (P et al., 1996). It is increasingly appreciated that DCM is more than a single-disease entity of “nonischemic” heart failure but rather represents a unique family of heart muscle diseases with complex interactions between genetic predisposition, infection, inflammation, autoimmune diseases, endocrine, and environmental precipitants (Asher et al., 2021).

Male sex is a key risk factor for progression to heart failure following a large number of cardiovascular conditions, including DCM (Cannata et al., 2020). Studies reported gender data for nongenetic DCM with an average overall sex ratio of 2.5:1, male to female (Jain et al., 2021). However, few clinical studies have specifically investigated gender-related differences in the incidence or pathogenic mechanisms of DCM. It was found that men with acute DCM had higher expression of apoptosis-related proteins than that of women and higher expression levels associated with lower left ventricular ejection fraction (LVEF; the fraction of the volume of fluid ejected from the left ventricle with each contraction) (Sheppard et al., 2005a).

The most common causes of DCM are infections and autoimmunity. Enteroviruses, adenoviruses, and herpesviruses are commonly found in patients with DCM (Maekawa et al., 2007). Virus infection triggers the recruitment of inflammatory cells including mast cells, macrophages, helper T cells, and B cells (Schultheiss et al., 2019). These immune cells release cytokines, such as transforming growth factor-β1 (TGFβ1), interleukin (IL), and tumor necrosis factor (TNF), and other mediators that promote remodeling, collagen deposition, and fibrosis (Epelman et al., 2015). In addition, a number of factors, including the components of innate immunity and profibrotic cytokines, have been identified in animal models as important pathogenic mechanisms that increase inflammation and susceptibility to chronic DCM (Elamm et al., 2012). Correspondingly, immunoadsorption with subsequent immunoglobulin substitution (IA/IgG) therapy could improve LVEF, LVIDD, and NYHA classes of DCM (Ameling et al., 2016).

With the rapid development of sequencing technologies, an increasing number of competing endogenous RNAs (ceRNAs), such as microRNA (miRNA), long noncoding RNA (lncRNA), and circular RNA (circRNA), have been found to be involved in DCM progression (Lin et al., 2021). The genes most commonly known to cause DCM, including TTN, LMNA, MYH7, BAG3, TNNT2, and others, were identified initially in large DCM pedigrees (Schultheiss et al., 2019). Interestingly, a number of circRNAs are generated from genes which are associated with cardiovascular diseases, such as TTN and RYR2 (Tan et al., 2017). In addition, emerging evidence reveals critical roles for lncRNAs in the development and progression of DCM (Chen et al., 2021). Moreover, downregulation of the miRNA-221/222 family associated with heart failure enables profibrotic TGF-β signaling in pressure-overloaded hearts (Verjans et al., 2018).

Historically, pharmacological therapy (ACE inhibitors and β-blockers) and cardiac resynchronization therapy (CRT) are standard treatments for heart failure in DCM, but they all have limitations. Recently, immunotherapies have become an attractive therapeutic strategy in DCM. Moreover, sex differences and gene expression influence the efficacy of immunotherapies (Jain et al., 2021). Therefore, to provide personalized immunotherapy for DCM patients, it is crucial to identify key genes and pathways that may be related to the phenotypic diversity of male and female patients. In our study, we used a GSE19303 gene expression microarray of the myocardial biopsy samples from DCM patients. First, we found that immunotherapy significantly improved the clinical outcome of male DCM patients. Furthermore, we identified the sex-related DEGs in DCM and constructed pathways and functional enrichment analysis. We identified sex difference immune–related ceRNA network with high reliability, and our results showed that the lncRNA–miRNA–mRNA network may provide a new understanding of the mechanisms and potential therapeutic targets for DCM.

## Materials and Methods

### Data Source

The GSE19303 gene expression microarray and clinical features dataset was obtained from Gene Expression Omnibus (http://ncbi.nlm.nih.gov/geo/). The dataset contained a total of 81 endomyocardial biopsy samples, 40 baseline biopsies from patients with DCM, 33 of 40 patients had received immunotherapy (immunoadsorption with subsequent immunoglobulin substitution (IA/IgG) treatment), 33 follow-up biopsies of DCM patients collected 6 months after the treatment, and eight biopsies from individuals without DCM (Ameling et al., 2016). Among them, 40 baseline and 33 follow-up endomyocardial biopsy samples from DCM patients were utilized in our study and were divided into groups of different sexes. The male patient group contained 28 samples, and the female patient group included 12 samples. The platform for the gene expression profiles was GPL570 [HG-U133_Plus_2] Affymetrix Human Genome U133 Plus 2.0 Array (Affymetrix; Thermo Fisher Scientific, Inc., Waltham, MA, United States).

### Research Design and Data Preprocessing

We retrieved the expression matrix from the GEO database and preprocessed it by using the robust multiarray analysis (RMA) method (http://www.bioconductor.org/). After log2 transformation and quantile normalization of the expression data, we annotated the converted probe ID for each gene to a gene symbol utilizing hgu133plus2. db, org. Hs.eg.db, and the annotate package in Bioconductor (http://www.bioconductor.org/). If a gene’s symbol corresponded with the multiple probe IDs, the expression level of that gene was represented by the mean of the probes.

### Clinical Features Analysis

T-tests or paired t-tests were used to test for differences, and outlier samples were assessed using 1.5 times the interquartile range of the differences, and Shapiro–Wilk normality test was used for normality tests. A *p*-value less than 0.05 is considered statistically significant.

### Immune Cell Infiltration Abundance Analysis

IOBR (Immuno-Oncology Biological Research) is a computational tool for immuno-tumor biology research (Zeng et al., 2021). Here, based on our expression profiles, we use the IOBR package in R to analyze the immune cell infiltration abundance of GSE19303 datasets. The xCell method was selected to calculate infiltration abundance of 64 kinds of immune cells, stem cells, and stromal cells in each sample (Aran et al., 2017). The DEGs of the ceRNA network were divided into the high-expression and low-expression groups by the median. The relationship between DEG expression and the fractions of immune cells was investigated by Wilcoxon test. The results were visualized using the ggplot packages in R software.

### Identification of Sex Difference Differentially Expressed Genes

The Linear Models for Microarray Analysis (Limma) package in R software was applied to identify the differentially expressed genes (DEGs) in the male DCM samples compared with the female DCM ones, based on Student’s t-test. Adjusted *p*-values were calculated using the Benjamini–Hochberg method. The significant DEGs were selected with a threshold of *p*-value < 0.05 and fold change>1.5. We obtained the volcano plot utilizing the pheatmap package in R.

Moreover, we excluded the genes in the Y chromosomes and then compared the male and female DCM samples again using the Limma package. *p*-value < 0.05 and fold change>1.5 were selected to be the cutoff criteria of the significant DEGs. We obtained another volcano and heatmap plot. In order to plot the differentially expressed lncRNAs (DE-lncRNAs), we used the pheatmap package in R to construct a heatmap.

### Functional Enrichment Analysis

In order to explore the potential functions and pathways that may be altered by the DEGs, we applied the clusterProfiler package in R to perform the functional and pathway enrichment analyses of the identified DEGs. The Gene Ontology (GO; http://www.geneontology.org/) database was used to determine the biological processes (BPs) that the DEGs may be involved in. In addition, according to the modified Fisher’s exact test, the Kyoto Encyclopedia of Genes and Genomes (KEGG; http://www.genome.jp/kegg/pathway.html) database was used for pathway enrichment analysis of the identified DEGs. The selection criteria for the significant GO terms and pathways were *p* < 0.05, and the number of enriched genes were (also called count) > 2. The plots were performed by the ggplot2 package in R.

### Gene Set Enrichment Analysis

GSEA software (version 3.0) and c2. all.v7.4. symbols.gmt subcollection were obtained from the GSEA website (http://software.broadinstitute.org/gsea/index.jsp) (Subramanian et al., 2005). We divided the samples into two groups by sex. The minimum gene set was 5 and the maximum gene set was 5,000, with 1,000 resampling. *p*-value < 0.05 (as needed) or FDR <0.25 (as needed) were considered statistically significant.

### Screening of Sex Difference Immune-Related Genes

Potential interactions between DE-lncRNAs and DE-miRNAs and between DE-miRNAs and DEGs were predicted using DIANA (https://diana.e-ce.uth.gr/lncbasev3) (Karagkouni et al., 2020) and ENCORI databases (https://starbase.sysu.edu.cn/index.php) (Li et al., 2014), respectively. Only the lncRNA–miRNA and miRNA-DEG interactors, that had an opposite expression trend, were used to construct the ceRNA network. The immune gene list was obtained from the Immunology Database and Analysis Portal (IMMPORT) database (http://www.immport.org/) (Bhattacharya et al., 2014). The Venny online tool was used to analyze the overlapping genes (http://jvenn.toulouse.inra.fr/app/example.html) (Bardou et al., 2014).

### ceRNA Network Enrichment Analysis

Coexpression patterns in 29 immune-related DEGs were analyzed using Pearson’s correlation coefficient, and the results were visualized using the heatmap packages in R software. Cytoscape software (version 3.8.2, https://cytoscape.org) was used to develop the ceRNA network. For gene set functional enrichment analysis, we used the GO and KEGG annotations of genes in the R package org. Hs.eg.db (version 3.1.0) as the background to map the genes to the background set using the R package clusterProfiler (version 3.14.3) to perform enrichment analysis to obtain gene set enrichment results. *p*-value < 0.05 was considered statistically significant.

### Predicted Protein–Protein Interaction Network Analysis

A protein–protein interaction (PPI) network, comprising 50 ceRNA network coexpression proteins, was constructed by GeneMANIA (http://genemania.org/) (Warde-Farley et al., 2010). These nodes represent genes that are closely related to the ceRNA network in terms of physical interactions, shared protein domains, predictions, colocalization, pathway, coexpression, and genetic interactions. We use NetworkAnalyst (version 3.0, https://www.networkanalyst.ca/) to carry out the heart (left ventricle)-specific PPI, TF-miRNA interactions, and protein–chemical interaction analysis on the ceRNA network coexpression module (Zhou et al., 2019). In these networks, the nodes represent individual genes/proteins/chemicals, while the edges which connect the nodes correspond to a known, curated interaction between a given pair of nodes.

### Tool Usage

All the statistical analyses were performed using R (version 3.6.4) or SPSS (version 19.0), and a *p*-value less than 0.05 is considered statistically significant. The plots were performed by R, Cytoscape software (version 3.8.2, https://cytoscape.org), or SangerBox tools (version 3.0, http://www.sangerbox.com/tool).

## Results

### Baseline Clinical Characteristics of Dilated Cardiomyopathy Patients

The RNA array data for a total of 40 DCM patients were acquired from the GES19303 dataset. The detailed baseline clinical features are listed in Supplementary Table S1. Among the 40 participants, 28 were male and 12 were female. DCM patient gender (male vs. female) was significantly correlated with age (52.2 ± 9.21 years vs. 45.42 ± 8.08 years, *p* = 0.032) and Epstein–Barr virus (EBV) infection (0/28 vs. 2/12, *p* = 0.027). However, gender was not significantly correlated with other clinical features such as LVEF, LVIDD, BMI, inflammation index, total virus infection, PVB19 infection, HHV6 infection, HSV1 infection, and IgG treatment.

### Immunotherapy Significantly Improves the Clinical Outcome of Dilated Cardiomyopathy Patients

**FIGURE 1:**
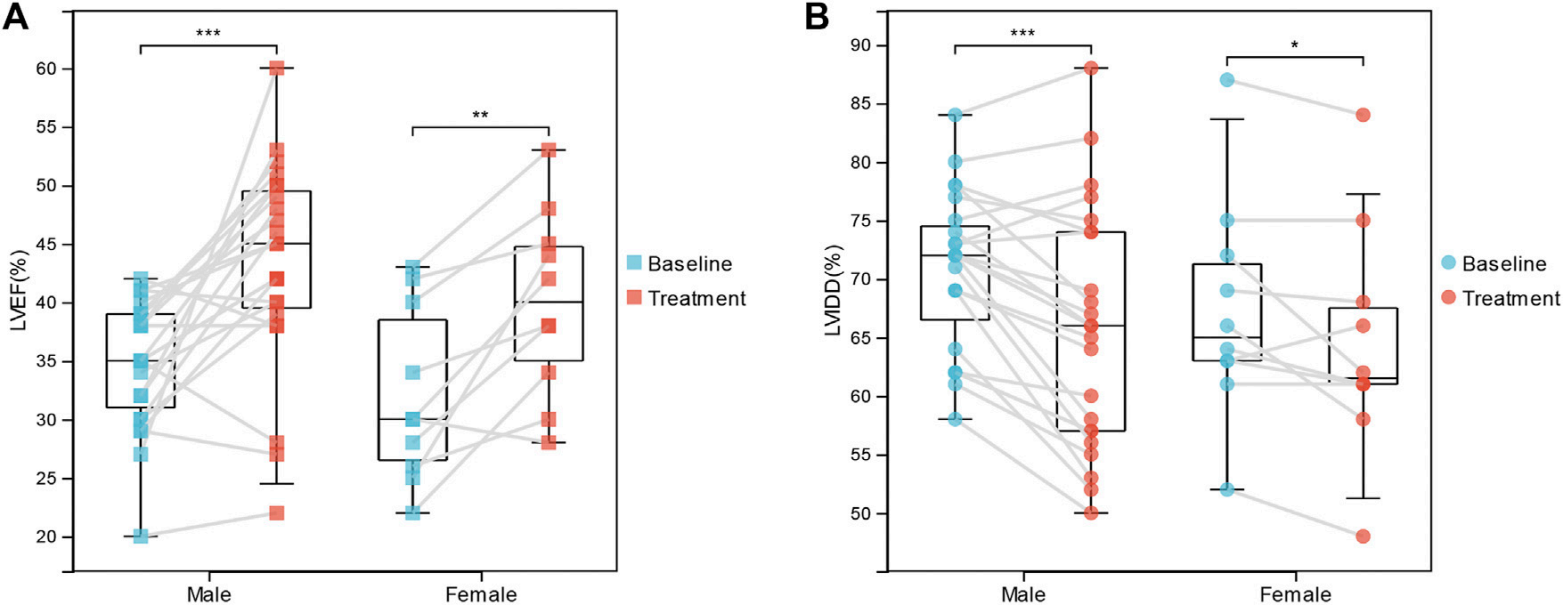
Correlations Between IgG immunotherapy and Clinical Outcome Features. **(A)** LVEF in paired baseline and IgG treatment DCM patients. **(B)** LVIDD in paired baseline and IgG treatment DCM patients. *, *p* < 0.05; **, *p* < 0.01; and ***, *p* < 0.001.

Moreover, we investigated the association between IA/IgG treatment and clinical outcome features in DCM patients. In female patients, immunotherapy was significantly correlated with LVEF (32.00 ± 7.44% vs. 40.00 ± 7.93%, *p* = 0.002) and LVIDD (67.20 ± 9.40% vs. 64.40 ± 9.79%, *p* = 0.047). For male patients, immunotherapy was more significantly correlated with LVEF (34.70 ± 5.43% vs. 43.22 ± 8.88%, *p* = 3.74E-04) and LVIDD (70.74 ± 6.72% vs. 65.70 ± 10.30%, *p* = 1.417E-04) (Figures 1A,B). However, IgG treatment was not significantly correlated with the inflammation index (Table 1). These results suggest that IA/IgG immunotherapy could significantly improve the outcome of DCM especially in male patients.

**TABLE 1:** Correlations between IA/IgG immunotherapy and clinical features of DCM in male and female patients.

Clinical Characteristics	Baseline	Follow up	*p* value
Male patients	—	—	—
LVEF (%)	34.70 ± 5.43	43.22 ± 8.88	**3.74E-04**
LVIDD (%)	70.74 ± 6.72	65.70 ± 10.30	**1.417E-04**
Inflammation index (CD68^+^ + CD3^+^) %	21.26 ± 12.17	17.87 ± 8.88	0.271
Female patients	—	—	—
LVEF (%)	32.00 ± 7.44	40.00 ± 7.93	**0.002**
LVIDD (%)	67.20 ± 9.40	64.40 ± 9.79	**0.047**
Inflammation index (cd68^+^ + cd3^+^) %	16.80 ± 5.55	13.70 ± 7.66	0.132
			

Bold values indicate *p* < 0.05.

### Sex is Correlated With Immune Infiltration Levels in Dilated Cardiomyopathy

**FIGURE 2:**
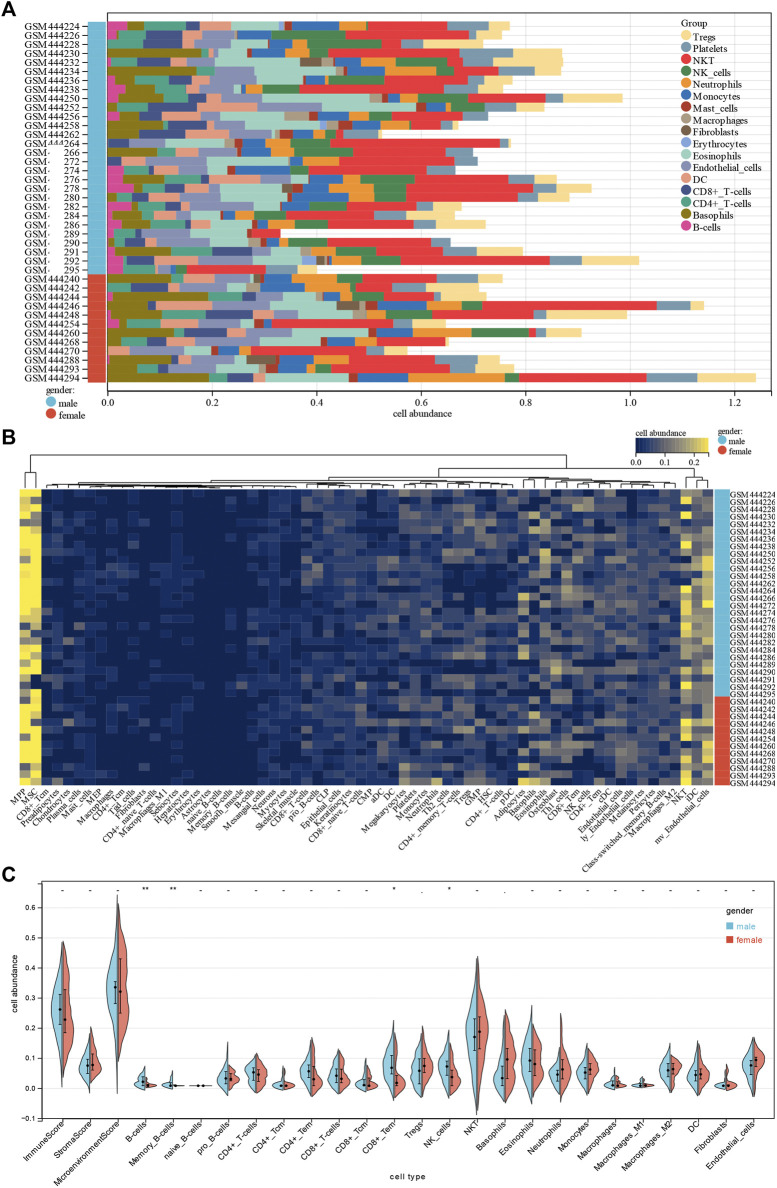
Correlations of gender with immune infiltration level in DCM tissues. **(A)** Distribution of immune cell infiltration in each sample. **(B)** Heatmap of immune cell types. **(C)** Violin plot of infiltrating immune cells.

To gain insight into potential target immune cells of DCM IA/IgG immunotherapy, we estimated the composition of the microenvironment in baseline DCM patients by using the xCell algorithm. Our result showed that the composition of the microenvironment of DCM was complex (Figure 2A). The top five abundant cell types were multipotent progenitors (MPPs), mesenchymal stem cells (MSCs), natural killer T cells (NKTs), immature dendritic cells (iDCs), and microvascular endothelial cells (mv endothelial cells) (Figure 2B). Moreover, when compared with the female DCM patients, male DCM patients had a significant positive correlation with abundance of B cells, memory B cells, effect memory CD8^+^ cells, and NK cells (Figure 2C). These results suggest that sex-differentiated microenvironments may contribute to differences in immunotherapy efficacy.

### Identification of Sex Difference Differentially Expressed Genes in Dilated Cardiomyopathy

**FIGURE 3:**
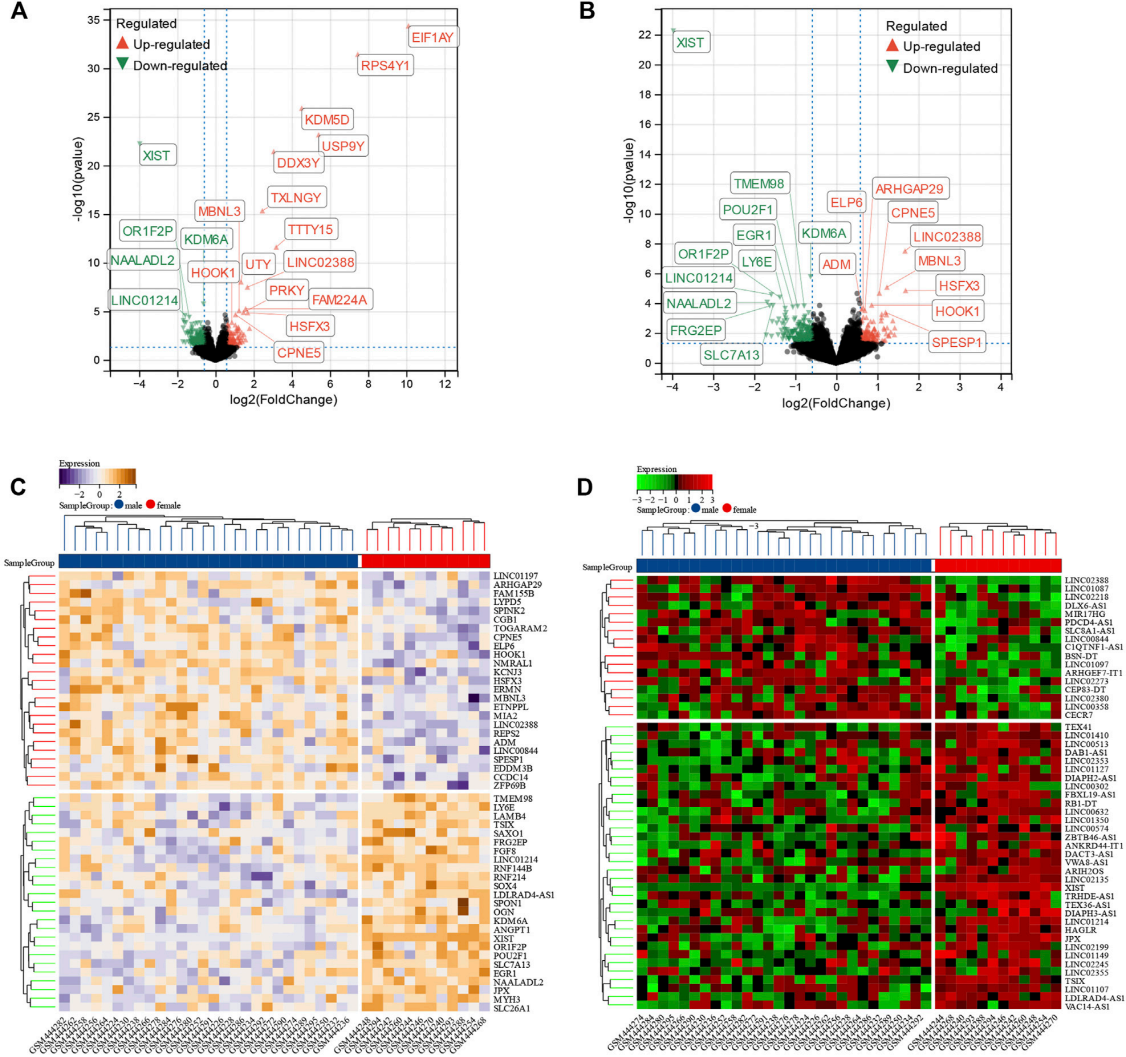
Identification of sex difference DEGs in DCM. **(A)** The volcano plot of all DEGs of male and female DCM patient myocardial tissues. The differences are set as *p* value<0.05 and |log FC| >1.5. **(B)** The volcano plot of all DEGs of male and female DCM patients after excluding the genes on Y chromosomes. The differences are set as *p* value<0.05 and |log FC| >1.5. **(C)** Heatmap showing 50 significantly DEGs of male and female DCM patients after excluding the genes on Y chromosomes. **(D)** Heatmap showing 50 significant DE-lncRNAs of male and female DCM patients after excluding the genes on Y chromosomes.

A total of 1,138 DEGs were finally screened from the comparison of male DCM samples with female DCM samples, including 579 upregulated and 556 downregulated DEGs (Figure 3A). Considering that genes in the Y chromosome is few in number and misleading, we removed the genes in the Y chromosome in the subsequent analyses. After excluding the genes in the Y chromosomes, we obtained 1,071 DEGs, of which 542 were upregulated and 529 were downregulated in the male group (Figure 3B). The top 50 significant positive and negative sex-related DEGs correlated with DCM are shown in the heatmap (Figure 3C). Interestingly, there were 68 differentially expressed lncRNAs (DE-lncRNAs) and five differentially expressed miRNAs (DE-miRNAs) in DEGs, and the top 50 significant DE-lncRNAs are shown in the heatmap (Figure 3D).

### Sex Difference Signaling Pathways and Functional Enrichment Analysis in Dilated Cardiomyopathy

**FIGURE 4:**
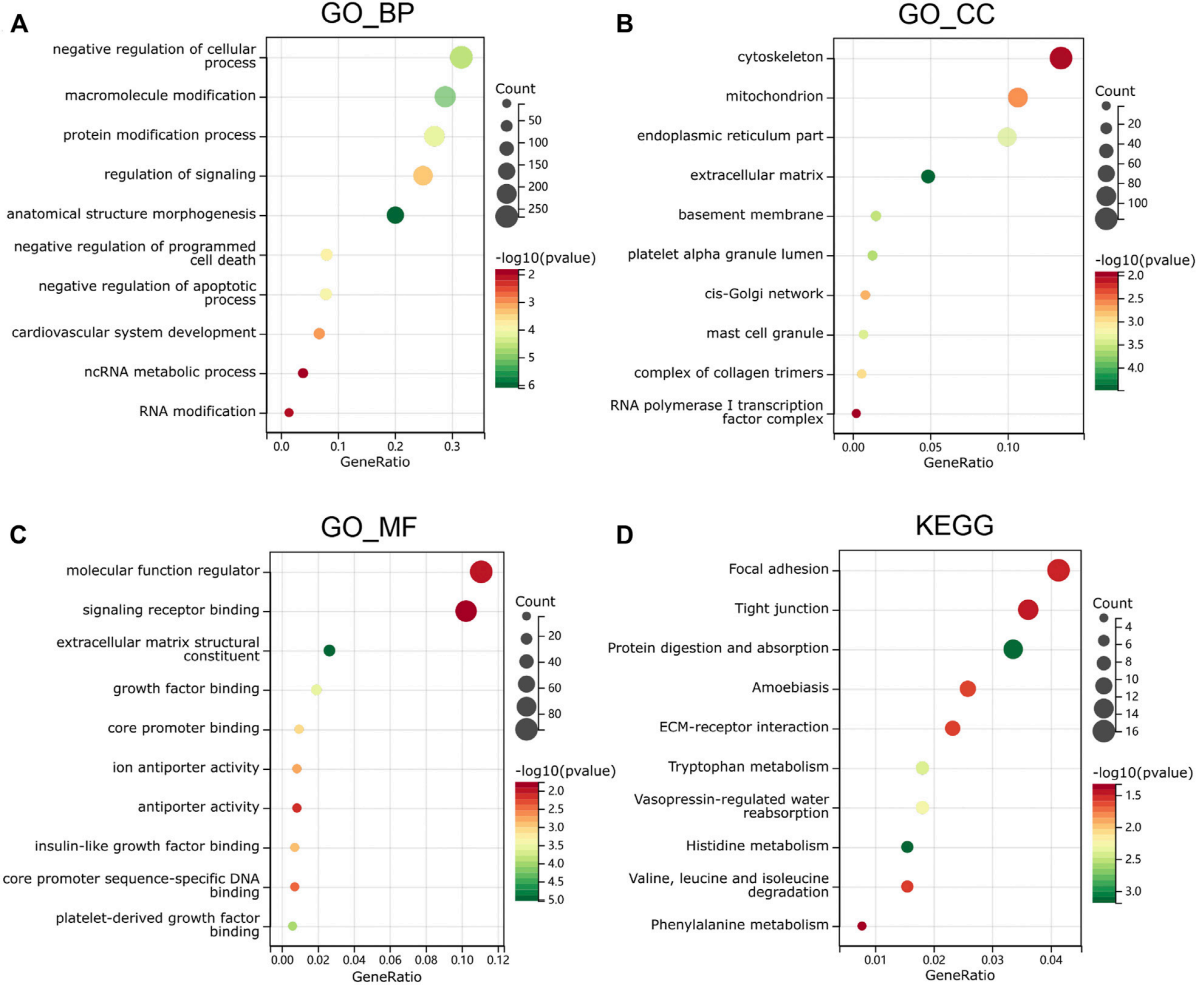
Functional enrichment analysis in GO and KEGG. **(A)** biological process, **(B)** molecular function and **(C)** cell composition of GO enrichment analysis. **(D)** Kyoto Encyclopedia of Genes and Genomes analysis results of differentially expressed gender-related genes between male and female. The words on the left indicates enriched terms, the size of the balls indicates the number of the genes enriched and the color indicates the level of the enrichment.

The upregulated and downregulated DEGs were processed separately for the GO and KEGG pathway analyses. The significantly enriched biological processes (BPs) were negative regulation of cellular process, negative regulation of programmed cell death, cardiovascular system development, ncRNA metabolic process, and RNA modification (Figure 4A). The significantly enriched cellular components (CCs) were the cytoskeleton, endoplasmic reticulum part, extracellular matrix, complex of collagen trimers, and RNA polymerase I transcription factor complex (Figure 4B). The significantly enriched molecular functions (MFs) were molecular function regulator, signaling receptor binding, extracellular matrix structural constituent, core promoter binding, and antiporter activity (Figure 4C). The significantly enriched KEGG were focal adhesion, tight junction, amebiasis, ECM–receptor interaction, and amino-acid metabolism (Figure 4D). These results suggest that there is a widespread impact of sex difference on the signaling transduction, transcriptional regulation, and metabolism.

**FIGURE 5:**
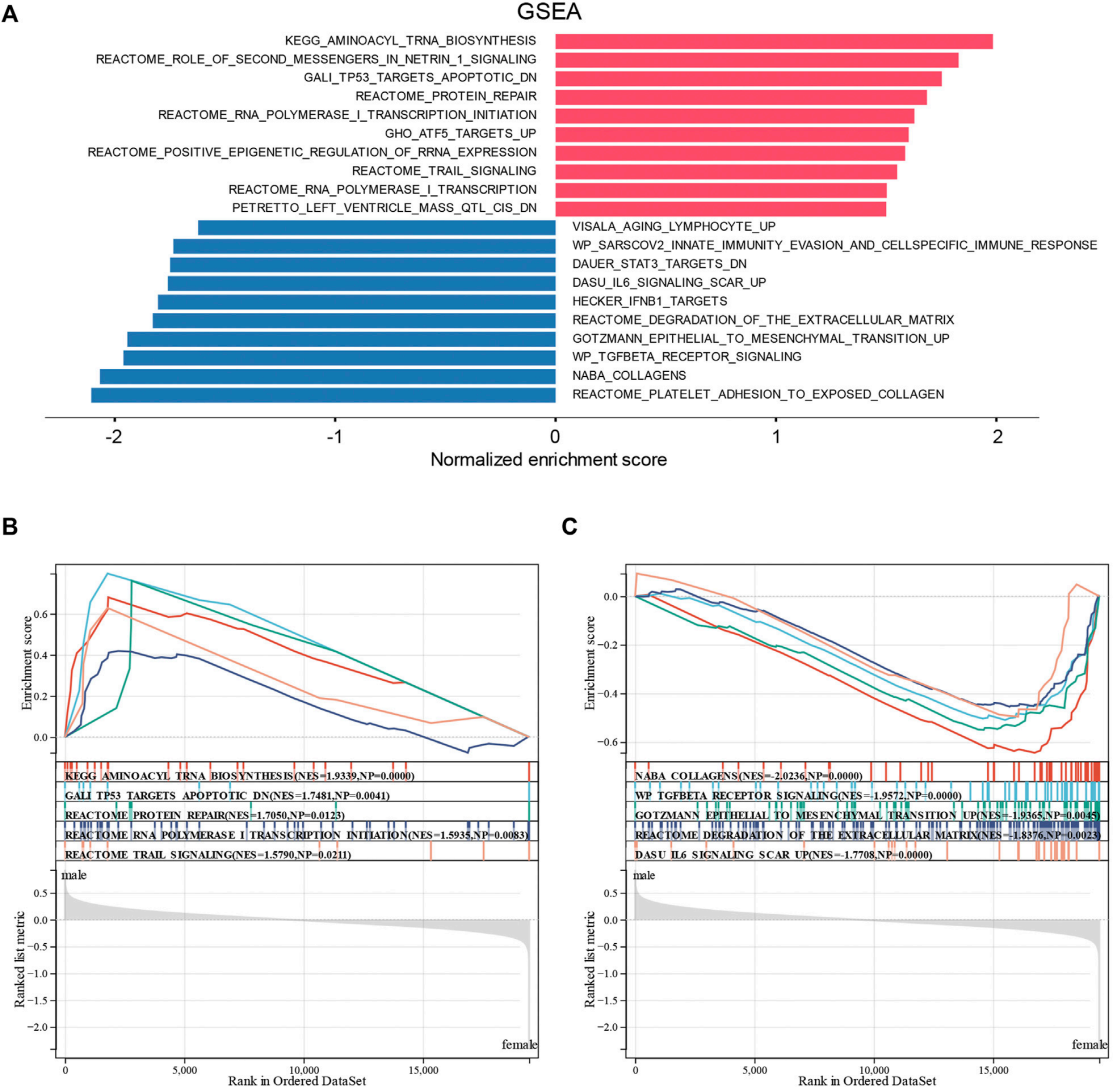
Enrichment plots from GSEA. **(A)** Significantly enriched pathway annotations of DCM. **(B)** Significantly positive enriched pathways in male DCM patients. **(C)** Significantly positive enriched pathways in female DCM patients.

To confirm the abovementioned results, we performed GSEA on the sex-related DEGs to identify Gene Ontology and signaling pathways that were differentially activated in DCM. The top 20 significant positive and negative enrichment pathways are shown in Figure 5A. GSEA showed that significant positively enriched pathways in male are aminoacyl tRNA biosynthesis, TP53 targets apoptotic, protein repair, RNA polymerase I transcription initiation, and TRAIL signaling. (Figure 5B). Gene sets related to collagens, TGF-beta receptor signaling, epithelial-to-mesenchymal transition, degradation of the extracellular matrix, and IL6 signaling showed enrichment in the female DCM patients (Figure 5C).

### Construction of Sex Difference Immune-Related ceRNA Network

**FIGURE 6:**
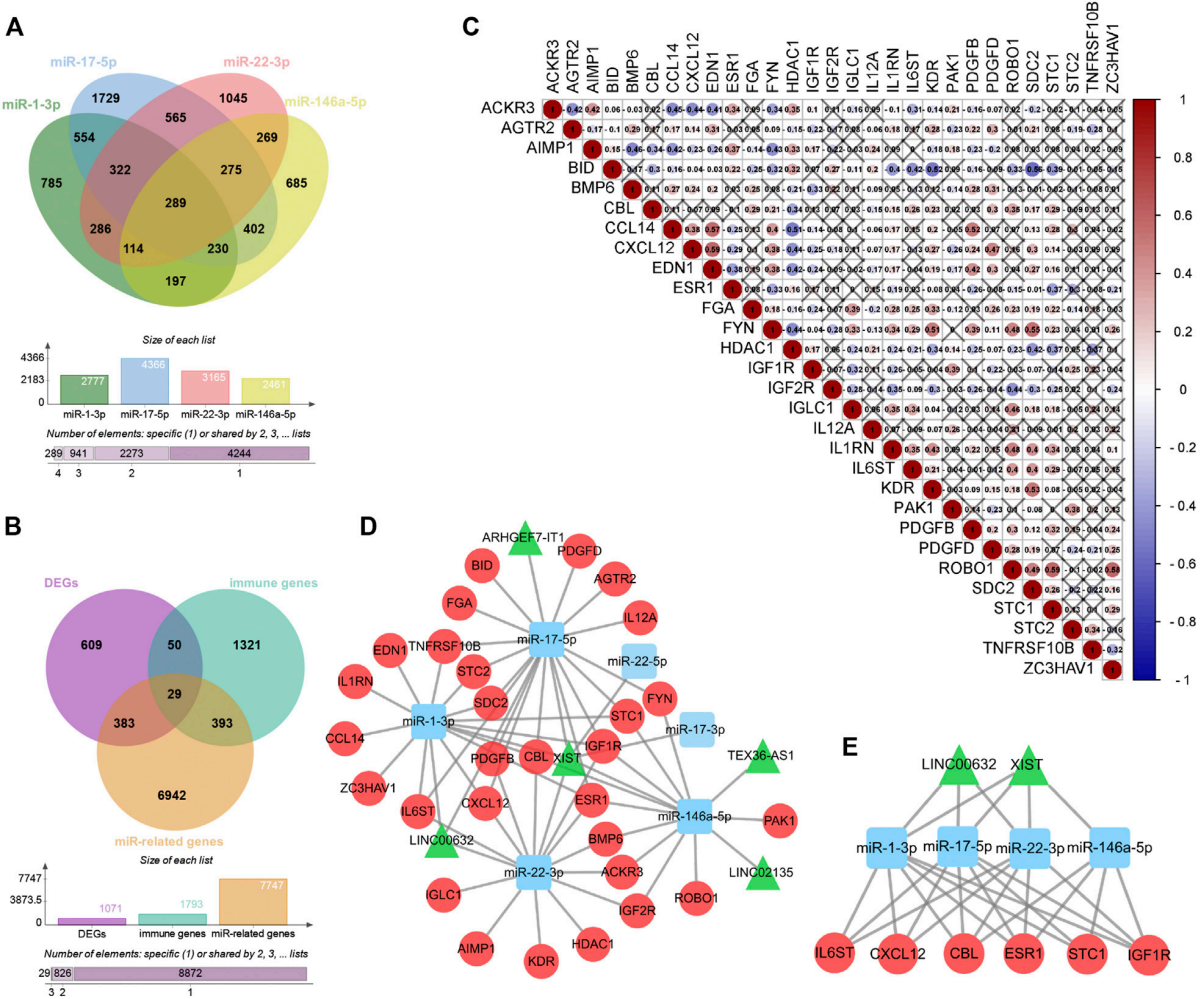
Construction of Sex difference Immune-related ceRNA Networks. **(A)** Intersection between four kinds of microRNAs. **(B)** Intersection between DEGs, miR-related genes and immune genes. **(C)** Co-expression analysis of immune-related DE-mRNAs. **(D)** ceRNA network among lncRNAs, miRNAs, and mRNAs, Circular, mRNAs; triangle, lncRNAs; square, miRNAs. **(E)** Central module from the ceRNA network.

Among the 68 DE-lncRNAs and five DE-miRNAs, five DE-lncRNAs (ARHGEF7-IT1, LINC00632, LINC02135, TEX36-AS1, and X-inactive specific transcript (XIST)) were predicted to interact with four DE-miRNAs (miR-1-3p, miR-17-5p, miR-22-3p, and miR-146a-5p) by the DIANA database. The four DE-miRNAs were predicted to interact with a total of 7,747 genes by the ENCORI database (Figure 6A). To further construct the sex difference immune-related ceRNA network, the Venny method was used to analyze the intersection between DEGs, miR-related genes, and immune genes (Figure 6B). The coexpression plot among these 29 genes is presented in Figure 6C. These DE-lncRNA–miRNA–immune gene interaction pairs were integrated to construct the ceRNA network (Figure 6D). Furthermore, we extracted a central module from the ceRNA network, including two lncRNAs (XIST and LINC00632), three miRNAs (miR-1-3p, miR-17-5p, and miR-22-3p), and six mRNAs (Casitas B-Lineage Lymphoma Proto-Oncogene (CBL), C-X-C Motif Chemokine Ligand 12 (CXCL12), Estrogen Receptor 1 (ESR1), Insulin-like Growth Factor 1 Receptor (IGF1R), Interleukin 6 Cytokine Family Signal Transducer (IL6ST), and Stanniocalcin 1 (STC1)) (Figure 6E).

**FIGURE 7:**
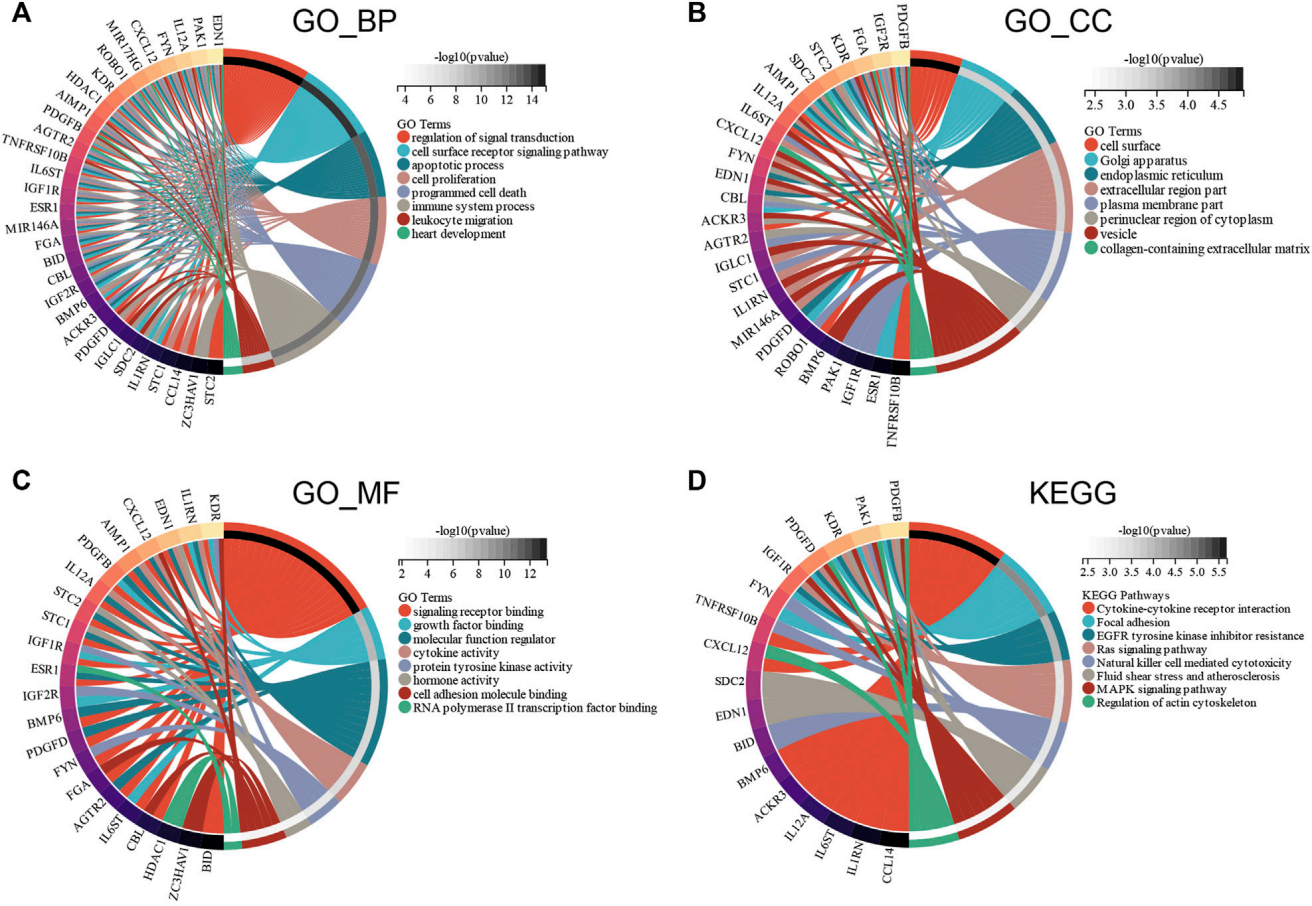
Functional enrichment analysis of ceRNA Network. **(A)** biological process, **(B)** molecular function, **(C)** cell composition and **(D)** KEGG analysis results of differentially expressed immune-related genes between male and female.

GO and KEGG enrichment analyses were conducted to investigate the functions of the ceRNA network. GO_BP analysis showed that ceRNA network was significantly enriched in programmed cell death, immune system process, and heart development (Figure 7A). GO_CC analysis showed that the ceRNA network was significantly enriched in the cell surface, endoplasmic reticulum, and collagen-containing extracellular matrix (Figure 7B). GO_MF analysis showed that the ceRNA network was significantly enriched in molecular function regulator, cytokine activity, and RNA polymerase II transcription factor binding (Figure 7C). The KEGG pathway enrichment analysis showed that ceRNA network was significantly enriched in cytokine–cytokine receptor interaction, Ras signaling pathway, and natural killer cell–mediated cytotoxicity (Figure 7D).

### Regulators of Sex Difference Immune-Related ceRNA Network in Dilated Cardiomyopathy

**FIGURE 8:**
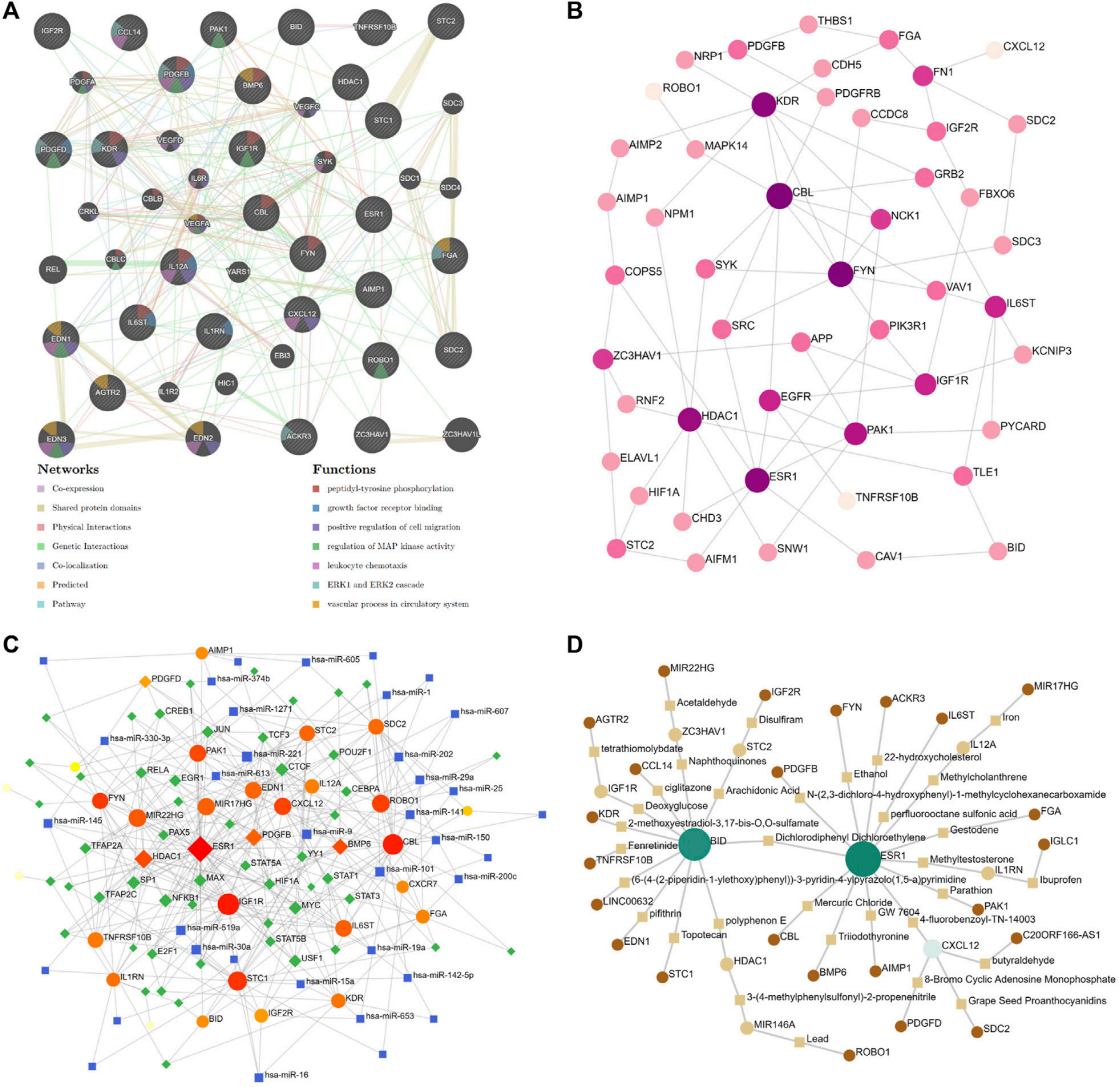
Significantly ceRNA networks in DCM. **(A)** 50 most significant co-expressed of protein-protein interaction (PPI) network by GeneMANIA. **(B)** heart-specific protein-protein interaction network by NetworkAnalyst. **(C)** transcription factor-miRNA (TF-miRNA) coregulatory interactions network by NetworkAnalyst. **(D)** Potential target drugs of sex difference immune-related ceRNA Network by NetworkAnalyst.

The protein–protein interaction (PPI) network was assembled based on the ceRNA network in the DCM cohort by GeneMANIA. The analysis showed that the 50 most significantly coexpressed genes play roles in peptidyl-tyrosine phosphorylation, leukocyte chemotaxis, and the vascular process in the circulatory system (Figure 8A). Next, the ceRNA network was assembled based on the heart (left ventricle)-specific data collected from the DifferentialNet database (Basha et al., 2018) by NetworkAnalyst (Figure 8B). The top five hub proteins were CBL, FYN Proto-Oncogene (FYN), Kinase Insert Domain Receptor (KDR), ESR1, and Histone Deacetylase 1 (HDAC1) (Supplementary Table S2). Furthermore, a graph of TF-miRNA coregulatory interactions of the ceRNA network was constructed based on the RegNetwork database (Liu et al., 2015) (Figure 8C). From this, the top five TFs identified were Myelocytomatosis Oncogene (MYC), Nuclear Factor Kappa B Subunit 1 (NFKB1), Specificity Protein 1 (SP1), MYC Associated Factor X (MAX), and Upstream Transcription Factor 1 (USF1) (Supplementary Table S3).

### Identifies Potential Target Drugs of Sex Difference Immune-Related ceRNA Network

To gain insight into the potential target drugs based on our established ceRNA network, we examined protein–chemical interactions from the Comparative Toxicogenomics Database (CTD) (Davis et al., 2021) (Figure 8D). Excluding hazardous chemicals, the top five drugs were 4-fluorobenzoyl-TN-14003, polyphenon E, 3-(4-methylphenylsulfonyl)-2-propenenitrile, deoxyglucose, and arachidonic acid (Supplementary Table S4). 4-fluoro benzoyl-TN-14003(BKT140, motixafortide) is a high-affinity CXCR4 antagonist, which can inhibit the migration of prostate cancer cells (Peng and Kopecek, 2014). BKT140 has been tested in stimulating megakaryopoiesis and platelet production (Abraham et al., 2013). Polyphenon E is a green tea polyphenol preparation which possesses potent antioxidative and anti-inflammatory properties (Bornhoeft et al., 2012). 3-(4-methylphenylsulfonyl)-2-propenenitrile (BAY11-7,082) is an NF-kappaB inhibitor, which can protect the myocardial infarction heart from cardiac dysfunction in the mouse model (Martinez-Martinez et al., 2017). Moreover, BAY11-7,082 significantly reduced the TNF and IL-6 protein expression in atherogenesis (Vallejo et al., 2018). Deoxyglucose (2-DG) is a glucose molecule which cannot undergo further glycolysis. 2-DG can antagonize DOX-induced cardiomyocyte death, which is mediated through multiple mechanisms, including the preservation of ATP content, the activation of AMPK, and the inhibition of autophagy (Chen et al., 2011). Arachidonic acid (AA) is an essential fatty acid, which can be found in fish and certain plant oils. Higher *in vivo* circulating and tissue levels of AA were associated with lower risk of major cardiovascular events (Marklund et al., 2019). In addition, sex differences in the AA levels could be an important underlying mechanism for different effects of sex hormones and cardiovascular disease differences between males and females (Gerges and El-Kadi, 2021). Based on the previous research, these drugs show promising potential as novel therapies against DCM *via* the immune-related ceRNA network. However, further evaluation is still needed.

### Correlation of Immune Cell Infiltration and ceRNA Network

**FIGURE 9:**
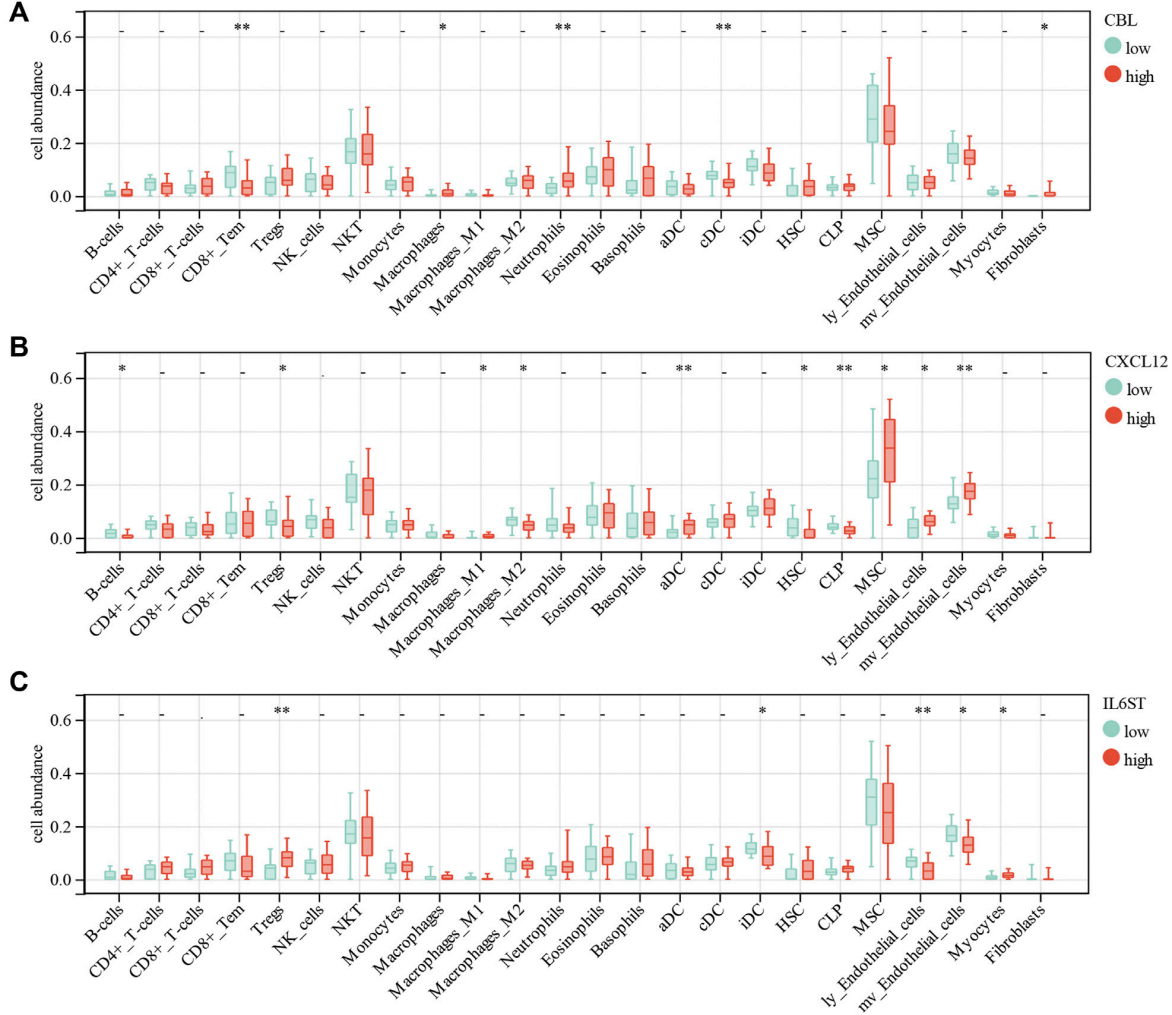
Correlations of low/high **(A)** CBL, **(B)** CXCL12 and **(C)** IL6ST expression with immune cells infiltration in DCM.

For further analysis, the DEGs of the ceRNA network were divided to the high-expression and low-expression groups by median. The correlation between immune cell abundance and DEGs was analyzed by the Wilcoxon test. CBL expression had a significant positive correlation with macrophages (*p* = 0.03), neutrophils (*p* = 0.008), and fibroblasts (*p* = 0.02). Moreover, CBL expression had a significant negative correlation with CD8+Tem (*p* = 0.0096) and conventional DC (cDC, *p* = 0.0056) (Figure 9A). CXCL12 expression had a significant positive correlation with M1 macrophages (*p* = 0.03), activated DCs (aDC, *p* = 0.0035), MSCs (*p* = 0.02), lymphatic endothelial cells (ly endothelial cells, *p* = 0.04), and mv endothelial cells (*p* = 0.0032). Furthermore, CXCL12 expression had a significant negative correlation with B-cells (*p* = 0.01), Treg (*p* = 0.03), M2 macrophages (*p* = 0.04), hematopoietic stem cells (HSC, *p* = 0.04), and common lymphoid progenitor (CLP, *p* = 0.0067) (Figure 9B). IL6ST expression had a significant positive correlation with Tregs (*p* = 0.0014) and myocytes (*p* = 0.03). Moreover, IL6ST expression had a significant negative correlation with iDC (*p* = 0.02), ly endothelial cells (*p* = 0.0044), and mv endothelial cells (*p* = 0.01) (Figure 9C). However, ESR1, IGF1R, XIST, and LINC00632 expression had no significant correlation with most immune cells in DCM.

## Discussion

The etiology of DCM can be broadly categorized into genetic, acquired, or mixed (Martinez et al., 2021). Susceptibility in the model systems to an elevated innate immune response is dependent on at least two factors: 1) male sex (e.g., testosterone) and 2) genetic background (Elamm et al., 2012). Males significantly more often developed relevant reduction of LVEF, malignant ventricular arrhythmias, and end-stage heart failure compared with females, and the mortality was also higher in males (Meyer et al., 2014). For a reason, these so-called sex hormones bind to the nuclear-associated receptors in cardiac cells such as cardiomyocytes and fibroblasts, where they influence cell function (e.g., apoptosis and fibrosis) (Sheppard et al., 2005b). Moreover, sex hormone receptors are located on/in many cells of the immune system, including T cells, B cells, monocytes, macrophages, DCs, and mast cells in humans, which activate the sex-specific immune response (Jain et al., 2021). With the intent to gain new genetic insight to understand the phenotypic differences between female and male patients with DCM, we performed this study and identified a set of critical DEGs which may facilitate therapeutic individualization. In this study, we obtained a total of 1,071 DEGs (542 upregulated genes and 529 downregulated genes) between the male and female DCM samples. The DEGs were processed separately for GO and KEGG pathway analyses. The most significantly enriched terms included “regulation of programmed cell death,” “cardiovascular system development,” and “ncRNA metabolic process.”

Viral infections of the heart were considered to be possible triggers or contributors for the development of the disease in a large number of the DCM patients (Kuhl et al., 2005). The most common viruses that induced inflammatory cardiomyopathy include adenoviruses and enteroviruses; vasculotropic viruses; human immunodeficiency virus (HIV), hepatitis C virus (HCV), influenza A virus and influenza B virus; and viruses from the Coronaviridae family and the Herpesviridae family (Tschope et al., 2021). Epstein–Barr virus belonging to the Herpesviridae family is ubiquitous in population and causes a latent, life-long B lymphocyte infection in more than 90% adults worldwide (Macsween and Crawford, 2003). The identification of intramyocyte EBV genome in inflammatory cardiomyopathy patients was up to 6.3% (Chimenti et al., 2004). Moreover, high numbers of EBV-encoded RNA copies were found in the CD8+T cells from endomyocardial biopsies of a female patient with life-threatening perimyocarditis and caused a severe chronic active infection (Richter et al., 2013). We found that DCM patient gender was significantly correlated with EBV infection. Both the sexes have the same seroprevalence of EBV. Nonetheless, EBV antibody titers in females are generally higher than those in males (Keane et al., 2021). Gender and sex hormone estradiol have been demonstrated to alter EBV latency III functions and regulate multiple sclerosis (MS) risk genes differently among both sexes (Keane et al., 2021). However, whether gender and sex hormones affect EBV infection in the myocardium of DCM patients still warrants further research.

In addition to sex differences, genetic predisposition plays a crucial role in the DCM process. The ceRNA network has been proven to be involved in various heart diseases, including DCM (Tao et al., 2019). In this study, we constructed an immune-related ceRNA network based on sex difference in DCM, including five lncRNAs, six miRNAs, and 29 mRNAs. Furthermore, we extracted a central module from the ceRNA network, including two lncRNAs (XIST and LINC00632), three miRNAs (miR-1-3p, miR-17-5p, and miR-22-3p), and six mRNAs (CBL, CXCL12, ESR1, IGF1R, IL6ST, and STC1). Recent studies found that XIST protects the hypoxia-induced cardiomyocyte injury by regulating different kinds of miRNAs (Chen et al., 2018; Fan et al., 2020; Xiao et al., 2021). LINC00632 inhibits IL-13–induced inflammatory cytokine and mucus production (Yue et al., 2020). Li et al. (2018) indicated that miR-1-3p, which correlates with the left ventricular function of HCM, can serve as a potential target and differentiate HCM from DCM. Zhao et al. (2021) reported that miR-17-5p–mediated endoplasmic reticulum stress promotes acute myocardial ischemia injury. Serially measured circulating miR-22-3p is a biomarker for adverse clinical outcome in patients with chronic heart failure (van Boven et al., 2017). CBL (c-Cbl) is an adapter protein with intrinsic E3 ubiquitin ligase activity that targets the receptor and nonreceptor tyrosine kinases, resulting in their ubiquitination and downregulation. Yang et al. (2016). demonstrated that c-Cbl mediates the ubiquitination/degradation of integrin β1, which leads to DCM. Rafiq et al. (2014) reported that c-Cbl activation promotes myocyte apoptosis, inhibits angiogenesis, and causes adverse cardiac remodeling after myocardial infarction. CXCL12, also known as stromal cell–derived factor-1 (SDF-1), plays a role in many diverse cellular functions, including embryogenesis, immune surveillance, and inflammation response. SDF-1β inhibits palmitate-induced cardiomyocyte fibrosis through the activation of the p38β MAPK signaling pathway (Tian et al., 2021). Jorbenadze et al. (2014) demonstrated that platelet-bound SDF-1 is especially increased in patients with severe impairment of left ventricular systolic function in heart failure. IL6ST, also known as gp130, is a signal transducer shared by many cytokines, including IL-6, ciliary neurotrophic factor (CNTF), leukemia inhibitory factor (LIF), and oncostatin M (OSM). Gp130 activation is sufficient to promote cardiomyocyte proliferation by activating Yap through Src during heart regeneration (Li et al., 2020). MiR-223-3p can directly combine with IL-6ST 3′ untranslatable regions (UTR) and hold back the IL-6 expression and decrease the expression of p-STAT3 and NF-κB p65 in Kawasaki-related heart disease (Wang et al., 2019).

Immune-targeted therapy has become an attractive therapeutic strategy in DCM recently. Damaged cardiac tissue and infections strongly induce the innate immune response, activating Toll-like receptors (TLRs) and the inflammasome, resulting in the release of the proinflammatory cytokines (Yajima and Knowlton, 2009). Immune cells needed for immune defense, such as macrophages, NK cells, and CD8 T cells, are also important in the early cardiac cellular response in the viral-related DCM models (Elamm et al., 2012). In the case of autoimmune DCM, B cells produce autoantibodies that form immune complexes with self-antigens and complement components (Liu et al., 2000). In addition, the proinflammatory markers, c-fos, IL-6, iNOS, and IL-1β, were upregulated only in the hearts of male but not female rats with autoimmune myocarditis (Barcena et al., 2021). Several clinical trials indicated that immunosuppressive therapies can significantly improve LVEF in patients with inflammatory DCM (Schultheiss et al., 2019). Ameling et al. (2016) demonstrated that immunoadsorption with subsequent immunoglobulin substitution (IA/IgG) improved LVEF, LVIDD, and NYHA classes and inflammation status in DCM patients, accompanied by lower expression of connective tissue growth factor, fibronectin, and collagen type I. However, the response rates to this therapeutic intervention are characterized by considerable interindividual variability (Ameling et al., 2013). Our results, partly in line with the findings of the previous studies, showed that the male sex was significantly positively correlated with inflammatory cell (B cells, memory B cells, CD8+Tem, and NK cells) infiltration.

In summary, in this comprehensive study, we found sex differences in the outcome of immunotherapy in DCM patients. In addition, male DCM patients had a significant positive correlation with the abundance of inflammatory cells (B cells, memory B cells, CD8^+^ Tem cells, and NK cells). Sex difference DEGs had a widespread impact on the signaling transduction, transcriptional regulation, and metabolism in DCM. Subsequently, we constructed an immune-related ceRNA network based on sex differences in DCM, including five lncRNAs, six miRNAs, and 29 mRNAs. This ceRNA network can regulate a variety of immune-related signaling pathways in DCM. Among this ceRNA network, CBL, CXCL12, and IL6ST were considered to be important DEGs associated with immune cell infiltration. Together, our findings suggest that the sex difference ceRNA network plays a crucial role in immune response regulation in DCM, yet the underlying mechanism still needed further validation.

## Data Availability

The original contributions presented in the study are included in the article/Supplementary Material. Further inquiries can be directed to the corresponding authors.
