# Supplementary material for: Construction of Immune-Related ceRNA Network in Dilated Cardiomyopathy: Based on Sex Differences
Source: Front Genet. 2022 Jun 8;13:882324. doi: 10.3389/fgene.2022.882324 (PMC9214033; doi:10.3389/fgene.2022.882324)
Supplement: Supplementary file 1 [file Table1.DOCX]

| Table 1. Correlations between IA/IgG immunotherapy and clinical features of DCM in male and female patients | | | | |
| --- | --- | --- | --- | --- |
| **Clinical characteristics** |  | baseline | follow up | *p* value |
| **Male patients** |  |  |  |  |
| LVEF (%) |  | 34.70±5.43 | 43.22±8.88 | **3.74E-04** |
| LVIDD (%) |  | 70.74±6.72 | 65.70±10.30 | **1.417E-04** |
| Inflammation index (CD68^+^ + CD3^+^) % |  | 21.26±12.17 | 17.87±8.88 | 0.271 |
| **Female patients** |  |  |  |  |
| LVEF (%) |  | 32.00±7.44 | 40.00±7.93 | **0.002** |
| LVIDD (%) |  | 67.20±9.40 | 64.40±9.79 | **0.047** |
| Inflammation index (cd68^+^ + cd3^+^) % |  | 16.80±5.55 | 13.70±7.66 | 0.132 |
| Bold values indicate *p*<0.05. |  |  |  |  |

| Table S1. Clinical characteristics of DCM patients in GSE19303. | | | |  |
| --- | --- | --- | --- | --- |
| **Clinical characteristics** |  | MALE(n=28) | FEMALE(n=12) | *p* value |
| Age (years) |  | 52.2±9.21 | 45.42±8.08 | **0.032** |
| LVEF (%) |  | 33.86±6.23 | 31.92±6.89 | 0.387 |
| LVIDD (%) |  | 71.25±7.15 | 66.42±9.13 | 0.080 |
| BMI |  | 28.41±4.95 | 26.65±3.36 | 0.269 |
| Inflammation index (cd68^+^ + cd3^+^) % |  | 19.89±11.62 | 17.17±5.11 | 0.442 |
| Virus infection | negative | 13 | 5 | 0.781 |
|  | positive | 15 | 7 |  |
| PVB19 | negative | 22 | 9 | 0.804 |
|  | positive | 6 | 3 |  |
| HHV6 | negative | 18 | 11 | 0.076 |
|  | positive | 10 | 1 |  |
| HSV1 | negative | 28 | 11 | 0.122 |
|  | positive | 0 | 1 |  |
| EBV | negative | 28 | 10 | **0.027** |
|  | positive | 0 | 2 |  |
| IA/IgG treatment | Yes | 23 | 10 | 0.928 |
|  | No | 5 | 2 |  |
| Bold values indicate *p*<0.05. | |  |  |  |

| Table S2. Genes of heart-specific protein-protein interaction network | | | |
| --- | --- | --- | --- |
| NCBI Entrez Gene ID | Label | Degree | Betweenness |
| 867 | CBL | 10 | 255.37 |
| 2534 | FYN | 9 | 223.7 |
| 3791 | KDR | 8 | 229.64 |
| 2099 | ESR1 | 8 | 211.89 |
| 3065 | HDAC1 | 7 | 168.6 |
| 5058 | PAK1 | 6 | 108.96 |
| 1956 | EGFR | 5 | 125.72 |
| 3480 | IGF1R | 5 | 106.21 |
| 3572 | IL6ST | 5 | 73.59 |
| 2335 | FN1 | 4 | 70.69 |
| 56829 | ZC3HAV1 | 4 | 58.51 |
| 4690 | NCK1 | 4 | 49.21 |
| 6850 | SYK | 3 | 67.87 |
| 3482 | IGF2R | 3 | 60.12 |
| 5295 | PIK3R1 | 3 | 52.35 |
| 351 | APP | 3 | 50.91 |
| 10987 | COPS5 | 3 | 48.56 |
| 2243 | FGA | 3 | 48.17 |
| 6714 | SRC | 3 | 47.94 |
| 7088 | TLE1 | 3 | 45.97 |
| 5155 | PDGFB | 3 | 35.22 |
| 2885 | GRB2 | 3 | 34.49 |
| 7409 | VAV1 | 3 | 27.14 |
| 8614 | STC2 | 3 | 20.5 |
| 1003 | CDH5 | 2 | 60.26 |
| 9672 | SDC3 | 2 | 53.02 |
| 1432 | MAPK14 | 2 | 47 |
| 4869 | NPM1 | 2 | 46.83 |
| 5159 | PDGFRB | 2 | 46.35 |
| 83987 | CCDC8 | 2 | 43.78 |
| 26270 | FBXO6 | 2 | 42.51 |
| 7965 | AIMP2 | 2 | 32.73 |
| 9131 | AIFM1 | 2 | 30.19 |
| 3091 | HIF1A | 2 | 24.99 |
| 857 | CAV1 | 2 | 22.23 |
| 6383 | SDC2 | 2 | 20.8 |
| 8829 | NRP1 | 2 | 20.37 |
| 6045 | RNF2 | 2 | 19.78 |
| 9255 | AIMP1 | 2 | 17.05 |
| 1107 | CHD3 | 2 | 14.43 |
| 30818 | KCNIP3 | 2 | 13.02 |
| 7057 | THBS1 | 2 | 10.5 |
| 22938 | SNW1 | 2 | 10.4 |
| 29108 | PYCARD | 2 | 9.45 |
| 1994 | ELAVL1 | 2 | 7.32 |
| 637 | BID | 2 | 6.66 |
| 8795 | TNFRSF10B | 1 | 0 |
| 6091 | ROBO1 | 1 | 0 |
| 6387 | CXCL12 | 1 | 0 |

| Table S3. TFs and miRNAs of TF-miRNA coregulatory interactions network | | | |
| --- | --- | --- | --- |
| NCBI Entrez Gene ID | Label | Degree | Betweenness |
| 2099 | ESR1 | 38 | 1830.06 |
| 3480 | IGF1R | 25 | 954.11 |
| 867 | CBL | 22 | 826.02 |
| 6781 | STC1 | 18 | 523.63 |
| 3065 | HDAC1 | 16 | 337.99 |
| 6091 | ROBO1 | 15 | 409.42 |
| 84981 | MIR22HG | 15 | 241.15 |
| 6387 | CXCL12 | 14 | 399.79 |
| 407975 | MIR17HG | 14 | 207.41 |
| 5058 | PAK1 | 13 | 340.44 |
| 3572 | IL6ST | 13 | 221.18 |
| 5155 | PDGFB | 13 | 191.88 |
| 1906 | EDN1 | 13 | 187.58 |
| 2534 | FYN | 12 | 500.77 |
| 654 | BMP6 | 11 | 326.09 |
| 8614 | STC2 | 11 | 178.56 |
| 6383 | SDC2 | 11 | 169.76 |
| 8795 | TNFRSF10B | 11 | 113.04 |
| 4609 | MYC | 9 | 254.33 |
| 4790 | NFKB1 | 9 | 217.91 |
| 10664 | CTCF | 9 | 192.61 |
| 6667 | SP1 | 9 | 174.76 |
| 3791 | KDR | 9 | 139.45 |
| 3592 | IL12A | 9 | 97.33 |
| 4149 | MAX | 8 | 168.17 |
| 7391 | USF1 | 8 | 147.11 |
| 3557 | IL1RN | 8 | 142.63 |
| 2243 | FGA | 8 | 94.08 |
| 3482 | IGF2R | 8 | 52.64 |
| 1958 | EGR1 | 7 | 146.63 |
| 3725 | JUN | 7 | 100.56 |
| 6772 | STAT1 | 6 | 131.55 |
| MIMAT0000278 | hsa-miR-221 | 6 | 80.13 |
| 57007 | CXCR7 | 6 | 77.29 |
| 9255 | AIMP1 | 6 | 75.95 |
| 3091 | HIF1A | 6 | 74.93 |
| 637 | BID | 6 | 71.96 |
| 7022 | TFAP2C | 6 | 62.32 |
| 5970 | RELA | 6 | 50.68 |
| 80310 | PDGFD | 6 | 46.88 |
| 7020 | TFAP2A | 6 | 39.39 |
| MIMAT0000441 | hsa-miR-9 | 5 | 92.75 |
| 6776 | STAT5A | 5 | 83.9 |
| 6777 | STAT5B | 5 | 82.48 |
| 5451 | POU2F1 | 5 | 80.64 |
| 1050 | CEBPA | 5 | 80.2 |
| 6774 | STAT3 | 5 | 78.66 |
| MIMAT0000099 | hsa-miR-101 | 5 | 75.03 |
| 5079 | PAX5 | 5 | 74.36 |
| 6929 | TCF3 | 5 | 59.84 |
| MIMAT0000087 | hsa-miR-30a | 5 | 57.04 |
| 1385 | CREB1 | 5 | 55.01 |
| MIMAT0002869 | hsa-miR-519a | 5 | 52.24 |
| 7528 | YY1 | 5 | 44.8 |
| 1869 | E2F1 | 5 | 35.09 |
| 3172 | HNF4A | 4 | 136.31 |
| 1499 | CTNNB1 | 4 | 62.09 |
| 7026 | NR2F2 | 4 | 50.35 |
| MIMAT0005796 | hsa-miR-1271 | 4 | 48.94 |
| 6773 | STAT2 | 4 | 44.77 |
| MIMAT0000432 | hsa-miR-141 | 4 | 44.2 |
| MIMAT0002811 | hsa-miR-202 | 4 | 42.34 |
| MIMAT0000069 | hsa-miR-16 | 4 | 40.35 |
| 6720 | SREBF1 | 4 | 39.04 |
| 5468 | PPARG | 4 | 36.2 |
| MIMAT0000086 | hsa-miR-29a | 4 | 32.97 |
| MIMAT0000437 | hsa-miR-145 | 4 | 32.26 |
| 367 | AR | 4 | 26.26 |
| 7157 | TP53 | 4 | 20.01 |
| 2353 | FOS | 4 | 17.17 |
| 6935 | ZEB1 | 4 | 17 |
| 571 | BACH1 | 3 | 126.95 |
| 25 | ABL1 | 3 | 47.64 |
| MIMAT0000451 | hsa-miR-150 | 3 | 44.36 |
| MIMAT0000751 | hsa-miR-330-3p | 3 | 36.22 |
| 6688 | SPI1 | 3 | 35.24 |
| 7704 | ZBTB16 | 3 | 33.28 |
| MIMAT0003275 | hsa-miR-607 | 3 | 31.97 |
| 2623 | GATA1 | 3 | 30.23 |
| MIMAT0004955 | hsa-miR-374b | 3 | 28.19 |
| MIMAT0000416 | hsa-miR-1 | 3 | 26.61 |
| MIMAT0000433 | hsa-miR-142-5p | 3 | 26.55 |
| MIMAT0000068 | hsa-miR-15a | 3 | 26.5 |
| MIMAT0003328 | hsa-miR-653 | 3 | 24.68 |
| MIMAT0000617 | hsa-miR-200c | 3 | 24.35 |
| 6721 | SREBF2 | 3 | 23.32 |
| MIMAT0003273 | hsa-miR-605 | 3 | 21.99 |
| MIMAT0000081 | hsa-miR-25 | 3 | 20.99 |
| 56829 | ZC3HAV1 | 3 | 20.21 |
| 6908 | TBP | 3 | 19.96 |
| 5454 | POU3F2 | 3 | 16.96 |
| 2113 | ETS1 | 3 | 14.49 |
| 6886 | TAL1 | 3 | 13.92 |
| 4087 | SMAD2 | 3 | 12.44 |
| MIMAT0003281 | hsa-miR-613 | 3 | 12.19 |
| 1051 | CEBPB | 3 | 11.93 |
| MIMAT0000073 | hsa-miR-19a | 3 | 9.75 |
| 4089 | SMAD4 | 3 | 7 |
| 186 | AGTR2 | 3 | 5.64 |
| 338917 | VSX2 | 2 | 118 |
| 5989 | RFX1 | 2 | 27.54 |
| MIMAT0000261 | hsa-miR-183 | 2 | 24.51 |
| MIMAT0003165 | hsa-miR-545 | 2 | 19.22 |
| MIMAT0003234 | hsa-miR-569 | 2 | 15.14 |
| MIMAT0003251 | hsa-miR-548a-3p | 2 | 14.89 |
| MIMAT0000727 | hsa-miR-374a | 2 | 14.23 |
| MIMAT0000423 | hsa-miR-125b | 2 | 14.17 |
| MIMAT0004701 | hsa-miR-338-5p | 2 | 13.39 |
| MIMAT0000758 | hsa-miR-135b | 2 | 12.63 |
| MIMAT0000274 | hsa-miR-217 | 2 | 12.15 |
| MIMAT0001541 | hsa-miR-449a | 2 | 11.85 |
| MIMAT0000770 | hsa-miR-133b | 2 | 9.68 |
| MIMAT0000763 | hsa-miR-338-3p | 2 | 8.29 |
| 4005 | LMO2 | 2 | 8.07 |
| 5915 | 5915 | 2 | 7.07 |
| 1386 | ATF2 | 2 | 4.97 |
| 6778 | STAT6 | 2 | 4.12 |
| 286411 | RP1-177G6.2 | 1 | 0 |
| 253868 | C20orf166-AS1 | 1 | 0 |
| 406938 | MIR146A | 1 | 0 |

| Table S4. Potential target drugs and genes of sex difference immune-related ceRNA Network | | | |
| --- | --- | --- | --- |
| NCBI Entrez Gene ID/  MeSH Unique ID | Label | Degree | Betweenness |
| 2099 | ESR1 | 13 | 1600 |
| 637 | BID | 11 | 1476 |
| 6387 | CXCL12 | 4 | 372 |
| D003633 | Dichlorodiphenyl Dichloroethylene | 2 | 1085 |
| C477728 | 4-fluorobenzoyl-TN-14003 | 2 | 413 |
| C472086 | polyphenon E | 2 | 305 |
| 3065 | HDAC1 | 2 | 248 |
| C434003 | 3-(4-methylphenylsulfonyl)-2-propenenitrile | 2 | 189 |
| D003847 | Deoxyglucose | 2 | 189 |
| D008748 | Methylcholanthrene | 2 | 189 |
| D008777 | Methyltestosterone | 2 | 189 |
| D009285 | Naphthoquinones | 2 | 189 |
| D016718 | Arachidonic Acid | 2 | 189 |
| 3480 | IGF1R | 2 | 128 |
| 3557 | IL1RN | 2 | 128 |
| 3592 | IL12A | 2 | 128 |
| 406938 | MIR146A | 2 | 128 |
| 56829 | ZC3HAV1 | 2 | 128 |
| 8614 | STC2 | 2 | 128 |
| C003585 | 22-hydroxycholesterol | 2 | 65 |
| C018475 | butyraldehyde | 2 | 65 |
| C020809 | tetrathiomolybdate | 2 | 65 |
| C033273 | Gestodene | 2 | 65 |
| C039671 | ciglitazone | 2 | 65 |
| C076994 | perfluorooctane sulfonic acid | 2 | 65 |
| C121565 | pifithrin | 2 | 65 |
| C401858 | GW 7604 | 2 | 65 |
| C451426 | N-(2,3-dichloro-4-hydroxyphenyl)-1-methylcyclohexanecarboxamide | 2 | 65 |
| C500344 | 2-methoxyestradiol-3,17-bis-O,O-sulfamate | 2 | 65 |
| C511402 | Grape Seed Proanthocyanidins | 2 | 65 |
| C516138 | (6-(4-(2-piperidin-1-ylethoxy)phenyl))-3-pyridin-4-ylpyrazolo(1,5-a)pyrimidine | 2 | 65 |
| D000079 | Acetaldehyde | 2 | 65 |
| D000431 | Ethanol | 2 | 65 |
| D004221 | Disulfiram | 2 | 65 |
| D007052 | Ibuprofen | 2 | 65 |
| D007501 | Iron | 2 | 65 |
| D007854 | Lead | 2 | 65 |
| D008627 | Mercuric Chloride | 2 | 65 |
| D010278 | Parathion | 2 | 65 |
| D014284 | Triiodothyronine | 2 | 65 |
| D015124 | 8-Bromo Cyclic Adenosine Monophosphate | 2 | 65 |
| D017313 | Fenretinide | 2 | 65 |
| D019772 | Topotecan | 2 | 65 |
| 186 | AGTR2 | 1 | 0 |
| 1906 | EDN1 | 1 | 0 |
| 2243 | FGA | 1 | 0 |
| 2534 | FYN | 1 | 0 |
| 253868 | C20ORF166-AS1 | 1 | 0 |
| 286411 | LINC00632 | 1 | 0 |
| 3482 | IGF2R | 1 | 0 |
| 3537 | IGLC1 | 1 | 0 |
| 3572 | IL6ST | 1 | 0 |
| 3791 | KDR | 1 | 0 |
| 407975 | MIR17HG | 1 | 0 |
| 5058 | PAK1 | 1 | 0 |
| 5155 | PDGFB | 1 | 0 |
| 57007 | ACKR3 | 1 | 0 |
| 6091 | ROBO1 | 1 | 0 |
| 6358 | CCL14 | 1 | 0 |
| 6383 | SDC2 | 1 | 0 |
| 654 | BMP6 | 1 | 0 |
| 6781 | STC1 | 1 | 0 |
| 80310 | PDGFD | 1 | 0 |
| 84981 | MIR22HG | 1 | 0 |
| 867 | CBL | 1 | 0 |
| 8795 | TNFRSF10B | 1 | 0 |
| 9255 | AIMP1 | 1 | 0 |
